# Mindfulness based art therapy study protocol to determine efficacy in reducing college stress and anxiety

**DOI:** 10.1186/s40359-021-00634-2

**Published:** 2021-09-03

**Authors:** Theresa Van Lith, Andrea Cheshure, Scott M. Pickett, Gregg D. Stanwood, Megan Beerse

**Affiliations:** 1 Department of Art Education, Florida State University, Tallahassee, FL USA; 2 School of Psychology and Public Health, La Trobe University, Melbourne, Australia; 3 Center for Translational Behavioral Science, Florida State University, Tallahassee, FL USA; 4 College of Medicine, Florida State University, Tallahassee, FL USA

**Keywords:** College students, Stress, Mindfulness, Mindfulness-based art therapy, Clinical trial, Intervention

## Abstract

**Background:**

College students in Generation Z are among the most stressed of our time. Previous research suggests that current interventions on university campuses are primarily for students in crises, but supportive services like psychoeducation to introduce coping skills are scant. Interventions that take both financial and time pressures into account are needed to address the mental health challenges faced by students. This study is designed to determine the unique role of the arts as a proactive mental health strategy for college students.

**Methods:**

A sample of college students in Generation Z (n = 120) will be recruited. Participants will be assigned to Arts-only, mindfulness-only, mindfulness-based art interventions or a non-intervention control group. These interventions will be delivered using a minimal contact, web-based approach. Participants will be screened for eligibility requirements prior to the inclusion in the Time 1 assessment though an online survey. Once enrolled, participants will complete the Time 1 assessment, followed by the intervention. Each assessment will consist of psychological and physiological measures. The MBAT, NCT and MO groups will complete a brief self-care task twice a week for 5 weeks. Upon completion of the assigned intervention, participants will complete a Time 2 assessment and participate in the Trier Social Stress Test. Six weeks post-intervention, participants will complete the final assessment to assess the longevity of effects of the intervention.

**Discussion:**

This study will clarify the effects of Mindfulness-based Art Therapy on several biometric and physiological markers above and beyond isolated art therapy or mindfulness interventions. Qualitative data in the form of transcribed exit interviews will be analyzed to characterize the unique needs of Generation Z students, along with level of engagement, intervention acceptance and satisfaction. The results will identify the efficacy of a low-cost and easily accessible mental health intervention targeting college students experiencing stress and anxiety.

*Trial registration* ClinicalTrials.gov, NCT04834765, 05/17/21. Retrospectively registered.

## Background

Generation Z make up nearly half (41%) of the total number of enrolled students at degree-granting universities in the U.S. [1]. They are also considered the most stressed generation of our time, with 91% of Gen Z respondents saying they have experienced at least one physical or emotional symptom due to stress in the last month compared to 74% of adults overall [2]. In the assessment of life stressors, 73% of respondents identified academics as the number one endorser of stressful periods. In the National Alliance on Mental Illness [3] survey, 73% of college student respondents reported having experienced at least one mental health crisis in college. Unfortunately, the increased burden placed on university counseling centers has forced universities to implement waitlists and limit the number of free sessions per student. Even after a student moves beyond the stigma of seeking help, they can experience a waiting time of 2–3 weeks before a counselor even sees them. After which, the counselor may determine that the student does not qualify for intensive intervention, and only offer group therapy or monthly sessions. This national mental health crisis has been getting attention over the last few years, as the Center for Collegiate Mental Health [4] identified the growing prevalence of student anxiety concerns as a substantial challenge for colleges and universities, and stated that developing supportive services to the general student body needs to be a top priority.

Currently, most mental health programming for college students focuses on interventions that are reactive in nature, and only address those in acute situations or crisis. According to the National Alliance on Mental Illness [3], American college students believe there is a need for mental health programs that provide psychoeducation about risk factors of anxiety, provide coping mechanisms for stress, and prioritize mental health at the same level as physical health. Additionally, strategies must be constructed to address the time restrictions and financial burden that college students experience. One possible modality to address this request is an online mindfulness based art therapy. Mindfulness-based Art Therapy (MBAT) is an approach that incorporates mindfulness practices like meditation and yoga into the practice of art therapy to promote health, wellness and adaptive responses to stress. MBAT deeply embodies the Bodymind model as it quite literally and concurrently activates body and mind processes. In this theoretical orientation, the emphasis is placed on the deeply interdependent relationship between the body and the mind; for example, neurocognitive research indicates that emotions are accessed through interoceptive and sensory mechanisms [5]. There is one standardized MBAT protocol that has been published to date and has demonstrated positive results in a real-world setting for women with breast cancer; these results include psychosocial adjustment and positive changes to cerebral blood flow [6–8]. MBAT-receiving participants demonstrated significant increases in blood flow at rest and during meditation in multiple limbic brain regions, including the insula, amygdala, hippocampus, and caudate nuclei. Moreover, responses to a stressful cue resulted in reduced activation of the posterior cingulate cortex in MBAT-receiving participants, demonstrating MBAT’s regulation of a specific neural circuit. Unfortunately, this MBAT protocol consists of 2-h sessions weekly for 8 weeks [9], which is more time- and cost-intensive than even CBT. Validation of more abbreviated and efficient MBAT approaches is crucial in determining whether this therapeutic approach can be effective in large-scale deployments. However, to validate this premise an investigation is required into the specific mechanisms at play in MBAT. While separately the literature supports both art and mindfulness for anxiety and stress management, the two mechanisms have not yet been studied where their combined effects are compared to their isolated effects. Therefore, our randomized-controlled research design is structured to tease apart the unique outcomes elicited by art and mindfulness separately, and determine if they have a super-additive relationship when combined.

This study is an extension of previous work [10, 11] with the addition of control groups to better understand the effects of MBAT on several psychological variables including stress and anxiety. We seek to determine the utility and biological substrates of a straightforward, reproducible, and easily deployable technology-assisted MBAT intervention on anxiety and related symptoms in young adults, college students in particular. Rising economic and academic pressures have exacerbated the prevalence of mood disorders among this group, but most college students still avoid traditional mental health services because of stigma. In preliminary studies, we have demonstrated the feasibility of a minimal contact, technology-delivered MBAT intervention. MBAT receiving students reported reduced anxiety and perceived stress as compared to controls performing a neutral-valence task. We also observed reductions in salivary stress hormone levels. In this project, we seek to leverage these intriguing data to validate a more intense MBAT intervention in a sensitive subpopulation with high initial anxiety. In the process, we will also use the Trier Social Stress Test (TSST) to experimentally induce a stress response and measure differences in reactivity, anxiety, and activation of the hypothalamic pituitary-adrenal axis. Saliva will be analyzed for multiple potential biological mediators including cytokines, inflammatory markers, and stress hormones. We predict that our MBAT intervention will produce sustained reduction of anxiety and perceived stress and reduced perceived stress responses to the TSST, mediated at least in part by reduced secretion of stress-related molecular signals.

To reach the general population of college students, a technology-assisted approach is paramount. This multidisciplinary project will tease apart the mechanisms of change and implications of employing art-based approaches for anxiety and stress reduction in college students at much more nuanced level than we have previously tested using the MBAT approach we designed and comparing it to its main components as isolated interventions. Based on our previous two studies and the accumulated research explored, the following study aims and hypotheses will guide our proposed study.

## Methods

The aims of this study are as follows:

1. To examine the psychological and physiological differences between an Mindfulness-based Art Therapy (MBAT) intervention, its components as isolated Art-Only (NCT) and Mindfulness-Only (MO) interventions, and a non-intervention control using a minimal contact, web-based approach with students in Generation Z.

### Hypothesis 1

MBAT, NCT, and MO will produce distinguishable differences in participant outcomes related to anxiety and stress symptomology, protective factors against chronic stress, and the biological stress response compared to a non-intervention control.

2. To evaluate how these interventions affect participants’ responses to an acute academic stress simulation using the TSST paradigm.

### Hypothesis 2

Results of self-report assessments and saliva samples collected during the acute academic stress simulation will produce distinguishable differences between group conditions, suggesting that the art-based interventions engage unique mechanisms of change compared to the Mindfulness-Only intervention.

3. To determine which intervention produces the greatest participant satisfaction and acceptance.

### Hypothesis 3

Participants of the MBAT and NCT interventions will report higher levels of study engagement, overall study satisfaction, and intervention acceptance compared to participants of the MO intervention and non-intervention control group conditions.

### Participants and eligibility criteria

This study will enroll a total of 120 students from Florida State University. To be eligible for the study, individuals must be enrolled in an academic program and fit within generation Z’s age category (18–26). Exclusion criteria for the study include (1) regular smoking (smoking is significantly associated with increased daily salivary cortisol release and (2) being over the age of 26.

### Recruitment

Participants will be recruited via emails to several departments within the university, associated social media and traditional flyers posted on campus. Participants will be informed of the eligibility criteria as required by the university’s Human Subjects Institutional Review Board (IRB). Interested students will be emailed a brief summary of the study and a consent form. Following confirmation of a signed consent, participants will be scheduled for an introductory in-person session wherein the study requirements will be explained.

### Procedure

Participants will be randomized into the experimental MBAT group condition, a neutral clay-manipulating task (NCT), a mindfulness only group (MO), or a control group condition. The study will be conducted over the course of 10 weeks within two academic semesters and used a minimal contact, technology assisted approach. The MBAT, NCT and MO groups are asked to complete a brief self-care task twice a week; these techniques were dubbed “self-care challenges” and were designed to only take 15 min. The MBAT group’s self-care challenges consisted of a 5-min mindfulness practice and 10 min of intentional art making with earth-based clay. The NCT group’s self-care challenges were identical from week to week, and consisted of a NCT, where they were given the same clay as experimental participants and told to “manipulate the clay in any way you wish for 15 min.” The MO group’s self-care challenges consisted of the same 5-min mindfulness practice as those in the MBAT group. The control group will participate in no self-care challenges during the 5 weeks. The study coordinator will meet with the individuals separately before Week 1 during which time quantitative data are collected in the form of saliva samples and a self-report questionnaire packet. For the 5 weeks in between face-to-face meetings, participants will check in through the study’s online platform, a learning management system, to complete the self-care challenges. Qualtrics© is used to administer the self-report assessments remotely. To demonstrate their continued participation in the study, participants assigned to the MBAT and NCT groups will upload images of their artwork each week within the online platform.

Each of the 10 modules is designed to take approximately 15–20 min and they are provided over the course of five weeks, a direct replication of our second pilot study, when preliminary data was gathered. From our first pilot study to our second, intervention delivery was condensed from once a week for 10 weeks to twice a week for five weeks, doubling our retention rate. All MBAT modules include a brief mindfulness practice of meditation, guided imagery, or yoga with a complementary clay-based art directive. The MO modules consist of only the mindfulness practices from the MBAT intervention. The NCT modules consist of an open-ended art-making exercise, with minimal direction. The NCT intervention is designed as an ‘art for art’s sake’ intervention with the focus on the art-making and less on outcome or product. All modules are completed through the online platform. Each of the MBAT modules are described in detail:

#### Modules 1 and 10

The first module is replicated on Week 5 to limit variables affecting biological outcome measures. Prior to the first meeting, participants will review and sign the consent form, register on the online platform, and filling out a demographic questionnaire and self-report assessments electronically. Clay kits with unique participant identification tags are distributed to participants of the MBAT and NCT groups as they arrive, which consist of a three-pound ball of natural clay in a sealed bag and instructions for caring for the clay.

#### Brief yoga sequence

Following the first face to face meeting, participants will follow along with the online five-minute light yoga sequence, where *asanas* (yoga postures) are cued with breath. Yoga, for the purposes of the study, is explicitly defined to participants as the unity of breath with body movement. Poses were chosen based on their accessibility to a variety of body types and abilities, and chosen because they are documented to aid in grounding, increased haptic awareness, lowered levels of stress and anxiety, and restoration.

#### Clay art directive

Following getting the participants more mindful of the present moment and bodily sensations through the yoga sequence, participants are asked to pull their ball of clay out and spend ten minutes creating a form from the clay to visually express their experience in the present moment. They are reassured that the final product of the art making will in no way be interpreted or judged; it is solely an exercise in mindfulness. Participants are then instructed to take a photograph of their art piece and upload the image to Canvas.

This same MBAT directive is facilitated through Qualtrics for module 10.

#### Module 2: introduction

Participants are asked to listen to the ‘Introduction’ meditation clip from Limbix©, which introduces the concept of mindfulness and meditation. Next, participants are asked to respond and spend 10 min creating a form from the clay that reflects the way they conceptualize mindfulness.

#### Module 3: using yoga

The ABM directive for week three consists of a four-minute yoga video practicing the ‘Breath of Joy’ yoga sequence that is commonly used for depression, anxiety, and stress. Next, the participants are asked to respond, being given the following instruction: “For the next 10 min, create a form out of your clay that reflects how your body feels *right now*.”

#### Module 4: releasing stress and anxiety

For this self-care challenge, participants are asked to begin with art making: “Create a piece out of your clay that represents what your stress and anxiety look/feel like (five minutes).” Next, they are to listen to the Limbix© meditation video titled “Releasing Stress and Anxiety.” After listening to the clip, they are asked to revisit their clay piece: “Did the breathing technique address your stress and/or anxiety? In what way(s)? Spend five minutes changing the piece based on your experience.”

#### Module 5: harnessing gratitude

Participants listen to the “Cultivate Gratitude” Limbix© video where they are directed to focus their attention on one specific thing for which they are grateful. For the art response, participants are asked to “create an art piece representing the specific thing you are grateful for.”

#### Module 6: revisiting yoga

For the second yoga video of the study, participants are guided through body alignment and a short yoga sequence consisting of extended mountain pose, forward fold to ragdoll, back up to extended mountain pose, ending in traditional mountain pose. Following the yoga video, they are asked to complete the art response: “For the next 10 min, create a form out of your clay that reflects how your body feels *right now*.”

#### Module 7: rejuvenate anytime

Participants listen to the Limbix video “Rejuvenate Anytime” for three minutes and then are asked to create an art response: “For the next 10 min, create a form out of your clay, keeping your attention on noticing how it feels in your hands.”

#### Module 8: restoration

Participants are provided a restorative yoga sequence consisting of a supported *Viparita Karani*, also known as ‘legs-up-the-wall pose,’ followed by supine twists. Participants are then asked to complete an art response: “For the next 10 min, create a form out of your clay that reflects how you feel *right now*.”

#### Module 9: build focus

For the module 9, participants are asked to begin with art making: “Create a form out of your clay reflecting how you are feeling right now.” After the art making, participants are to “Focus on [their] created form while listening to the [Limbix© “Build Focus”] clip.”

At the end of the 5-week intervention period, all participants will complete a final face to face meeting. During this session participants will go through an acute academic stress simulation- the Trier Social Stress Test (TSST) protocol. Trier Social Stress Test (TSST) is a validated paradigm to induce psychological stress in participants in a laboratory setting. It consists of 3 components. The first is an anticipation period of 10 min, where participants will prepare a speech for 3 judges that are part of the research team. The next two test components consist of delivering the prepared speech and completing a mental arithmetic exercise for the judges. The TSST will be used to experimentally induce a stress response and measure differences in reactivity, anxiety, and activation of the hypothalamic–pituitary–adrenal axis. The TSST has reliably produced heightened cortisol levels, above baseline levels [12]. Saliva will be collected at baseline on Week 1 of the intervention, before and twice immediately after the TSST. It will be analyzed for multiple potential biological mediators including cytokines, inflammatory markers, and stress hormones. Following the simulation, participants provide two final saliva samples and are given access to an exit survey and offered the opportunity to provide subjective feedback on their perception of the program’s effectiveness, user-friendliness, and any open-ended comments they would like to provide.

**Fig. 1:**
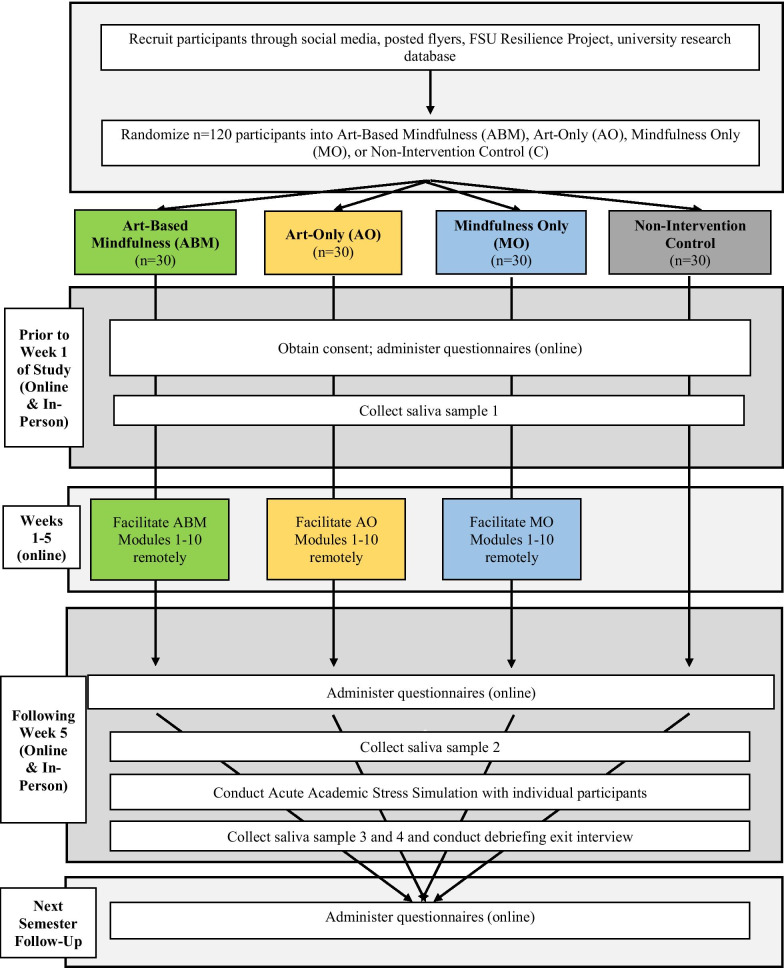
Study procedures

The self-report measures will be administered again 6 weeks later as a follow-up to allow us to discern whether beneficial effects are maintained. For full study procedures, please see Fig. 1.

### Measures

#### Questionnaire packet

Participants will complete a packet of questionnaires 3 times during this study. They will be given after the initial in-person session, immediately upon completion of the intervention modules and 6 weeks post intervention. The measures included are as follows:

Individual characteristics of participants including age, gender, ethnic background, major, and year in school will be collected via a demographic assessment administered after the initial in-person meeting via Qualtrics. In this demographic assessment, female participants will be asked the date of their last menstrual cycle to make sure 5th week cortisol sampling pairs with a similar timeline, as hormone concentrations are affected by menstruation cycles and can alter cortisol results.

Generalized Anxiety Disorder 7-item (GAD-7) is a self-report measure used to assess symptoms related to generalized anxiety during the past 2 weeks. Items assess the severity of symptoms including feelings of restlessness, excessive worry, trouble relaxing and nervousness/irritability on a 4-point Likert-type scale where a higher sum indicates severe anxiety.

Perceived Stress Scale 10-item (PSS-10) is a self-report measure of an individual’s levels of stress through assessing lack of control, unpredictability and overload over the past month. Items are on a 5-point Likert-type scale with high scores indicating higher perceived stress.

Patient Health Questionnaire 9-item (PHQ-9) is a brief self-report measure of depression severity. Scores range from 0 (not at all) to 3 (nearly every day) for items describing common symptomology associated with depression over the past two weeks. A sample item is “how often have you been bothered by little interest or pleasure in doing things”. Total scores indicate a range from minimal to a severe level of depression.

Somatic Symptom Scale (SSS-8) is a self-report measure of somatic symptom burden. Items are clustered into four categories: gastrointestinal, pain, cardiopulmonary and fatigue. Items are rated on a 5-point Likert-type scale and refer to symptomology over the past week.

Oldenburg Burnout Inventory (OLBI) is a self-report measure used to assess job burnout. This scale was adapted by the authors to assess academic burnout. This scale uses a two-factor structure to measure burnout through scores of disengagement and exhaustion, wherein higher scores indicate higher levels of burnout.

Five Facet Mindfulness Questionnaire-Short Form (FFMQ-SF) is a 24-item scale used to measure various aspects of mindfulness. An example item is “I find myself doing things without paying attention.” Each item is rated on a scale ranging from 1 to 5. This questionnaire uses a five-facet structure that includes non-reactivity, observation, description, acting aware and non-judgement. These scores are summed to create an overall mindfulness score.

Pittsburgh Sleep Quality Index (PSQI) is a 19-item self-report measure of sleep quality over the past month. This measure consists of seven components: subjective sleep quality, sleep latency, sleep duration, habitual sleep efficiency, sleep disturbances, use of sleep medication, and daytime dysfunction. These component scores can be summed to create an overall sleep quality score ranging from 0 to 21 with higher scores indicating poor sleep quality.

#### Biological measures

Human Interleukin-6 and cortisol saliva samples will be collected in adherence to the Salimetrics© saliva specimen preparation protocol. Participants will be asked to avoid alcohol consumption for 12 h and not to consume food for at least 1 h prior to sample collections. Water will be provided to participants to rinse their mouths thoroughly 10 min before each saliva sample is collected. Saliva samples will be collected by unstimulated passive drool.

Participants will be instructed to tilt their heads forward, allowing the saliva to pool on the floor of the mouth and then pass the saliva through their lips into a collection vial. Time and date of sample collections will be recorded, and samples will be collected at controlled times due to the diurnal variation in cortisol levels. Samples will be immediately placed on wet ice in a portable biohazardous cooler and then transported to the lab within three hours of collection and frozen at − 80 °C. Circulating interleukin-6 is a validated biomarker of generalized anxiety disorder.

### Data plan

Data will be analyzed by repeated measures ANOVA with time as a repeated measure and treatment arm as a between-subjects factor. Power analysis and previous experience indicates that sample size will be sufficient to reveal medium-sized effects (f = 0.3, power = 0.8). We expect the MBAT intervention will reduce severity of these reports from baseline to week 10 (after 5 weeks of intervention), particularly for the GAD-7 and PSS10 (based on preliminary data). We also predict that the MBAT intervention will diminish the impact of the TSST on anxiety and stress. Data obtained through the SSS-8 may be of particular interest in determining if the intervention improves systemic function, including anxiety-related gastrointestinal, cardiovascular, and musculoskeletal related systems. Our follow-up 6 weeks later, at approximately the same time in the semester as the initial measurements, will allow us to discern whether the expected beneficial effects of the intervention are persistent.

## Discussion

Our innovative intervention allows for easy scale-up. Furthermore, our results will allow us to develop a cost-effective MBAT approach that can be deployed quickly and consistently across multiple populations and health conditions with comorbid anxiety. The studies contained in this proposal will help us understand the biological and psychological mechanisms by which mindfulness-based art therapy (MBAT) can reduce mood disorder symptoms and physiological stress responses in college students. Using an innovative electronic delivery system for the MBAT protocol, we are able to advance our scientific understanding of the impact of a pragmatic, real-world mental health intervention through rigorous biobehavioral analysis.

## Data Availability

Requests of the datasets obtained from this study will be reviewed by the second and third author.

## Abbreviations

FFMQ-SF: Five Facet Mindfulness Questionnaire-short form
GAD-7: Generalized Anxiety Disorder-7
MBAT: Mindfulness-based Art Therapy
MO: Mindfulness only
NCT: Neutral Clay Task
OLBI: Oldenburg Burnout Inventory
PHQ-9: Patient Health Questionnaire-9
PSQI: Pittsburgh Sleep Quality Index
PSS-10: Perceived Stress Scale-10
SSS-8: Somatic Symptom Scale-8
TSST: Trier Social Stress Test
